# Health-Related Quality of Life in People Living with HIV andHypertension: Effects of a Short Message Service-Based Mobile HealthIntervention (MOPHADHIV Trial)

**DOI:** 10.1007/s10461-026-05128-z

**Published:** 2026-04-13

**Authors:** Danleen James Hongoro, Andre Pascal Kengne, Nasheeta Peer, Kim Nguyen, Olufunke A. Alaba

**Affiliations:** 1 Health Economics Unit, Faculty of Health Sciences, School of Public Health, University of Cape Town, Cape Town, South Africa; 2 Non-Communicable Diseases Research Unit, South African Medical Research Council, Cape Town, South Africa; 3 Department of Medicine, Faculty of Health Sciences, University of Cape Town, Cape Town, South Africa; 4 African Population and Health Research Center, Nairobi, Kenya

**Keywords:** HIV, Hypertension, Multimorbidity, Mobile health, Short message service, Health-related quality of life, VIH, Hipertensión, Multimorbilidad, mHealth, Calidad de vida relacionada con la salud, Intervención por SMS

## Abstract

Mobile health (mHealth) interventions offer scalable support for adherence and self-management, yet their effects on health-related quality of life (HRQoL) among people living with HIV (PLWH) and comorbid hypertension remain unclear. We evaluated HRQoL outcomes using data from the MOPHADHIV trial, a 12-month short message service (SMS)-based randomized controlled trial conducted at four community health centres in Cape Town, South Africa. HRQoL was measured using the EuroQol 5-Dimension 5-Level (EQ-5D-5L) utility index (Ugandan value set) and the EuroQol Visual Analogue Scale (VAS) at baseline and 12 months. Between-group differences were assessed using two-sample t-tests and analysis of covariance (ANCOVA) models adjusting for baseline HRQoL and covariates. A total of 582 participants (279 control; 303 intervention) with complete HRQoL data at baseline and 12 months were included in the analysis. Baseline HRQoL was high and comparable across arms. At 12 months, EQ-5D-5L utility was slightly higher in the control arm (0.97 vs 0.95; mean difference 0.03; t = 2.66, p = 0.01), corresponding to a small effect size (Cohen’s d = 0.22). In adjusted models, intervention allocation was associated with a small reduction in EQ-5D-5L utility (β = − 0.02, p = 0.01; fully adjusted β = − 0.03, p < 0.01). VAS scores improved in both groups, with no significant between-arm differences. In this cohort of PLWH with hypertension, a 12-month SMS-based intervention did not improve HRQoL and was associated with a small reduction in EQ-5D-5L utility. High baseline HRQoL and limited sensitivity of EQ-5D-5L to HIV-related psychosocial domains may partly explain these findings.

## Resumen

Las intervenciones de salud móvil (mHealth) ofrecen apoyo escalable para la adherencia y el autocuidado; sin embargo, sus efectos sobre la calidad de vida relacionada con la salud (CVRS) en personas que viven con VIH (PVVIH) y comorbilidad de hipertensión siguen siendo poco claros. Evaluamos los resultados de CVRS utilizando datos del ensayo MOPHADHIV, un ensayo controlado aleatorizado de 12 meses basado en mensajes de texto (SMS), realizado en cuatro centros de atención primaria en Ciudad del Cabo, Sudáfrica. La CVRS se midió mediante el índice de utilidad EuroQol 5-Dimension 5-Level (EQ-5D-5L) (con el conjunto de valores de Uganda) y la escala visual analógica EuroQol (VAS) al inicio y a los 12 meses. Las diferencias entre grupos se evaluaron mediante pruebas t de dos muestras y modelos de covarianza (ANCOVA) ajustados por valores basales y covariables. Un total de 582 participantes (279 control; 303 intervención) con datos completos de CVRS en ambos momentos fueron incluidos en el análisis. La CVRS basal fue alta y comparable entre grupos. A los 12 meses, la utilidad EQ-5D-5L fue ligeramente mayor en el grupo control (0.97 vs 0.95; diferencia media 0.03; p = 0.01), lo que corresponde a un tamaño de efecto pequeño (d de Cohen=0.22). En los modelos ajustados, la asignación a la intervención se asoció con una pequeña reducción en la utilidad EQ-5D-5L. Las puntuaciones VAS mejoraron en ambos grupos, sin diferencias significativas entre ellos. En esta cohorte de PVVIH con hipertensión, una intervención basada en SMS durante 12 meses no mejoró la CVRS en comparación con la atención habitual.

## Introduction

Living with Human Immunodeficiency Virus/Acquired Immunodeficiency Syndrome (HIV/AIDS) presents challenges beyond clinical management, with major implications for health-related quality of life (HRQoL) [1–5]. Poor HRQoL can hinder achievement of the UNAIDS 95-95-95 targets by affecting adherence, retention in care, and sustained engagement with treatment [6]. As survival improves, people living with HIV (PLWH) are increasingly experiencing age-related non-communicable diseases, particularly hypertension and other cardiovascular conditions [7–12].

The relationship between HIV, antiretroviral therapy (ART), and cardiovascular disease (CVD) risk is complex and multifactorial. First, traditional CVD risk factors such as aging, obesity, and lifestyle patterns remain important drivers of hypertension among PLWH, similar to the general population [10, 12–14]. Second, independent of treatment effects, HIV infection itself is associated with elevated cardiovascular risk through persistent immune activation and chronic systemic inflammation, even among individuals who are virally suppressed on ART [8, 9]. This inflammatory milieu contributes to endothelial dysfunction and atherosclerotic processes, thereby increasing CVD risk beyond traditional factors. Third, certain ART drug classes have been linked to metabolic changes. For example, integrase strand transfer inhibitors have been associated with weight gain and modest increases in hypertension incidence in some populations; however, these effects are generally small and not uniformly observed across all regimens or settings [8, 15]. Thus, hypertension among PLWH cannot be attributed solely to ART exposure but reflects the interplay of traditional risk factors, HIV-related inflammatory processes, and, in some cases, specific pharmacologic effects.

The coexistence of HIV and hypertension complicates care through increased pill burden, potential drug interactions, and cumulative cardiovascular risk [7–11, 15]. Beyond biomedical concerns, managing dual chronic conditions may exacerbate anxiety, treatment fatigue, and perceived health decline, all of which can negatively affect HRQoL. Monitoring HRQoL is therefore essential, as it captures dimensions of wellbeing not reflected in viral suppression or blood pressure control alone [16, 17]. Yet, traditional vertical care models often insufficiently address the multidimensional needs of PLWH with multimorbidity, including psychosocial functioning and daily activity limitations [18–20].

Mobile health (mHealth) interventions have emerged as promising strategies to strengthen chronic disease management in resource-limited settings [21–24]. These tools leverage mobile technologies to deliver remote, scalable support [25–27], ranging from SMS reminders to interactive, tailored feedback systems [28–31]. By providing private and flexible communication channels, mHealth interventions may reduce barriers related to stigma, transport constraints, and fragmented care delivery [27]. Evidence supports their effectiveness in improving adherence and engagement across HIV and cardiometabolic conditions [32–35], yet their impact on HRQoL among PLWH with comorbid hypertension remains underexplored.

This study addresses this gap by evaluating HRQoL changes among participants in the MOPHADHIV trial, a 12-month SMS-based intervention designed to support adherence and integrated chronic disease management. Using the EuroQol five-dimension five-level instrument (EQ-5D-5L) and the EuroQol Visual Analogue Scale (VAS), we assessed changes in subjective health status in this population. While the main trial outcomes focused on adherence and blood pressure control, incorporating HRQoL provides complementary insight into patient-centred outcomes. Clinical indicators measure biomedical effectiveness, but HRQoL reflects lived experience, functional status, and perceived wellbeing critical dimensions in the long-term management of HIV and hypertension.

## Methods

### Study Setting and Population

**Fig. 1:**
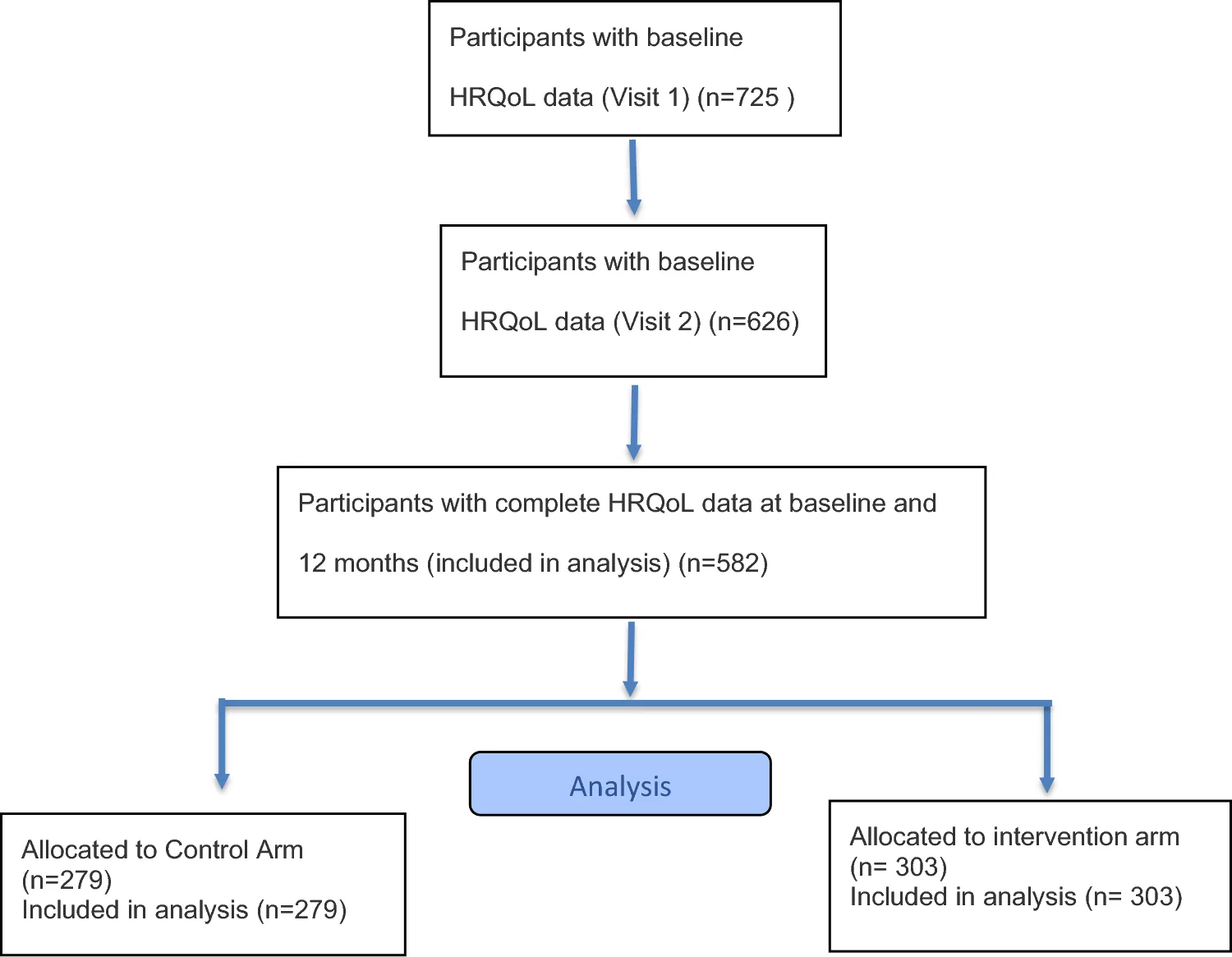
Flow of participants included in the HRQoL analysis from baseline (visit 1) to 12-month follow-up (visit 2)

Participant flow through each stage of the trial, including screening (n = 714), follow-up (n = 626), and inclusion in the final analysis (n = 582), is presented in the CONSORT flow diagram (Fig. 1).

This study was conducted between 2021 and 2024 at four community health centres in the Western Cape, South Africa, Mitchells Plain CDC, District Six CHC, Mfuleni CHC, and Kraaifontein CHC. Participants were adults living with HIV (PLWH) who were receiving antiretroviral therapy (ART) and were concurrently being treated for hypertension. All participants were enrolled as part of the MOPHADHIV randomized controlled trial.

### MOPHADHIV Trial and Study Aims

The MOPHADHIV trial was an mHealth intervention study designed to evaluate the effectiveness of mobile phone text messaging in promoting medication adherence and improving hypertension care outcomes among individuals with comorbid HIV and hypertension. The intervention targeted adults attending HIV treatment centres and aimed to support adherence to antihypertensive treatment through automated SMS reminders. The intervention consisted of standardised, one-way SMS messages, including medication reminders, general health promotion content, and encouragement to engage with routine care. Messages were brief and focused on reinforcing daily medication-taking behaviour and health awareness. Illustrative examples included reminders to take medication, encouragement messages highlighting the importance of adherence for long-term health, and prompts to seek care if unwell. These examples reflect the general content and tone of the intervention rather than verbatim messages.

The MOPHADHIV trial was a parallel, two-arm randomized controlled trial with 1:1 allocation. The trial is reported in accordance with the CONSORT 2010 guidelines[36]. Participants were enrolled from four HIV clinics in Cape Town. Eligible participants were adults living with HIV receiving antiretroviral therapy who were either receiving or eligible for treatment for hypertension. Randomization was conducted using a centralized web-based system employing non-deterministic minimization to ensure balance across study arms. Allocation was concealed from study personnel until assignment. Due to the nature of the intervention, participants and care providers were not blinded; however, outcome assessors and data analysts were blinded to treatment allocation until completion of the primary analysis. The trial was registered with the Pan African Clinical Trials Registry (PACTR201811878799717), available via the registry (https://pactr.samrc.ac.za).

This analysis focused on baseline and 12-month follow-up HRQoL data collected during the MOPHADHIV trial to examine changes over time and differences between the intervention and control groups. Although the intervention primarily targeted medication adherence, it was hypothesised that SMS support could influence HRQoL domains by improving routine functioning (usual activities), reducing symptom burden through better hypertension control (pain/discomfort), and providing reassurance that may influence anxiety or depression. However, as the intervention consisted of standardised, one-way messages, its potential to influence psychosocial domains may have been limited.

### Measurement of HRQoL

HRQoL was assessed using the EQ-5D-5L instrument and the VAS. The EQ-5D-5L captures health status across five domains mobility, self-care, usual activities, pain/discomfort, and anxiety or depression each with five severity levels. Responses were converted into a single utility index score using the Ugandan value set. At the time of analysis, no South African tariff was available; the Ugandan tariff [37], was selected as the most contextually relevant sub-Saharan African alternative. Utility scores were computed according to the general valuation formula:

$${U}_{it}=1-\sum_{d=1}^{5}\sum_{k=2}^{5}{\beta }_{dk}{D}_{dk,it}$$

where ${U}_{it}$ denotes the utility score for individual *i* at time *t*, ${\beta }_{dk}$ represents the tariff coefficient associated with level *k* in domain *d*, and ${D}_{dk,it}$ is a binary indicator equal to 1 if the individual reports level *k* in domain *d*. Utility scores were generated at baseline (${U}_{i0}$) and at 12 months (${U}_{i1}$). Participants also rated their overall health using the VAS, ranging from 0 (“worst imaginable health”) to 100 (“best imaginable health”), at baseline (${V}_{i0}$) and at 12 months (${V}_{i1}$).

### Covariates

Sociodemographic and household covariates were included to improve precision in adjusted models. These comprised age (continuous), gender (female vs male), education level (secondary vs primary), employment status (employed vs not working), household size, number of children, and indicators for electricity in the household, flush toilet access, and piped water access. Employment status was collapsed into a binary variable, where employed included full-time, self-employed (formal or informal), part-time, temporary, and casual work, and not working included unemployed, housewife, pensioner, student, disabled, and other non-working categories.

### Statistical Analysis

All analyses were conducted using *Stata version 17*. Two-tailed tests were used with a significance level set at 0.05. Complete-case analysis was applied for the primary regression models. Participants with missing EQ-5D-5L utility or VAS values at either baseline or 12 months were excluded from the relevant analyses. A complete-case indicator was constructed to identify participants with full HRQoL data at both time points. Baseline characteristics were compared between complete and incomplete cases using t-tests for continuous variables and chi-square tests [38], for categorical variables to assess potential attrition bias. Descriptive statistics were calculated for baseline and 12-month utility and VAS scores by treatment arm. Change scores were computed as:

$$\Delta U_{i} = U_{i1} - U_{i0}$$

$$\Delta V_{i} = V_{i1} - V_{i0}$$

Between-group differences in baseline, follow-up, and change scores were assessed using two-sample t-tests.

### Econometric Specification

The primary analytic approach was analysis of covariance (ANCOVA) [39–41], which estimates intervention effects at follow-up while adjusting for baseline values. For EQ-5D-5L utility, the following model was estimated:

$${U}_{i1}=\alpha +{\beta }_{1}{T}_{i}+{\beta }_{2}{U}_{i0}+{\varepsilon }_{i}$$

where ${U}_{i1}$ is the 12-month utility score, ${T}_{i}$ is a binary indicator equal to 1 for intervention and 0 for control, ${U}_{i0}$ is the baseline utility score, and ${\varepsilon }_{i}$ is an error term. The coefficient ${\beta }_{1}$ represents the adjusted treatment effect. For VAS outcomes, an analogous specification was used:

$${V}_{i1}=\alpha +{\gamma }_{1}{T}_{i}+{\gamma }_{2}{V}_{i0}+{\nu }_{i}$$

where ${V}_{i1}$ is the 12-month VAS score and ${V}_{i0}$ is the baseline VAS score. Fully adjusted models were then estimated by including sociodemographic and household covariates:

$$U_{i1} = \alpha + \beta_{1} T_{i} + \beta_{2} U_{i0} + {X^{\prime}}_{i} {\boldsymbol{\theta}} + \varepsilon_{i}$$

$$V_{i1} = \alpha + \gamma_{1} T_{i} + \gamma_{2} V_{i0} + {X^{\prime}}_{i} {\boldsymbol{\delta}} + \nu_{i}$$

where ${\mathbf{X}}_{i}$ denotes the vector of covariates and ${\boldsymbol{\theta}}$ and ${\boldsymbol{\delta}}$ are corresponding parameter vectors. All models were estimated using ordinary least squares (OLS).

### Standardised Effect Size

To facilitate interpretation of the magnitude of the intervention effect on EQ-5D-5L utility at 12 months, Cohen’s *d* was calculated as:

$$d = \frac{{\overline{U}_{control} - \overline{U}_{{{\mathrm{int}} ervention}} }}{{S_{p} }}$$

where the pooled standard deviation ${s}_{p}$ is defined as:

$${s}_{p}=\sqrt{\frac{{s}_{0}^{2}+{s}_{1}^{2}}{2}}$$

and ${s}_{0}^{2}$ and ${s}_{1}^{2}$ represent the variances in the control and intervention groups, respectively.

### Ethical Considerations

The MOPHADHIV trial, from which the HRQoL data for this analysis were drawn, was conducted in accordance with the Declaration of Helsinki and received ethical approval from the South African Medical Research Council (SAMRC) Human Research Ethics Committee (Protocol ID: EC006-2/2017; amendment approval granted on 23 April 2021). The present HRQoL analysis received separate ethical approval from the Human Research Ethics Committee of the University of Cape Town (HREC reference number 284/2022; approval granted on 23 June 2022). Written informed consent was obtained from all participants prior to enrolment.

## Results

### Sample and Treatment Allocation

A HRQoL data were available for 714 participants at baseline (Visit 1) and for 626 participants at 12-month follow-up (Visit 2). Complete EQ-5D-5L utility and VAS data at both time points were available for 582 participants, who were included in the HRQoL analysis dataset. Of these, 279 (47.94%) were allocated to the control arm and 303 (52.06%) to the intervention arm, indicating a near-balanced distribution across study groups (Table 1). The present analysis therefore reflects a complete-case analysis of participants with HRQoL data available at both baseline and 12 months.

**Table 1:** Treatment arm allocation (N = 582)

Treatment arm	N	%
Control	279	47.94
Intervention	303	52.06
Total	582	100.00

Complete-case availability for EQ-5D-5L utility and VAS at baseline and 12 months was 100.00% (582/582)

### EQ-5D-5L Utility and VAS at Baseline and 12 Months, by Treatment Arm

At baseline, mean EQ-5D-5L utility scores were high and similar across arms (Control: 0.95; Intervention: 0.94). Baseline VAS scores were also comparable between groups (Control: 69.35; Intervention: 69.50). At 12 months, mean EQ-5D-5L utility increased in both arms, reaching 0.97 in the control group and 0.95 in the intervention group (Table 2). Similarly, VAS scores improved over time in both groups, increasing to 77.16 in the control arm and 76.23 in the intervention arm. Overall, both EQ-5D-5L utility and VAS scores improved from baseline to 12 months in both treatment arms, with similar baseline values and slightly higher 12-month mean scores observed in the control group.

**Table 2:** EQ-5D-5L utility and VAS at baseline and 12 months, by arm (N = 582)

Outcome	Control mean (SE)	95% CI	Intervention mean (SE)	95% CI
EQ-5D-5L utility (baseline)	0.95 (0.01)	0.94, 0.96	0.94 (0.01)	0.92, 0.95
EQ-5D-5L utility (12 months)	0.97 (0.00)	0.96, 0.98	0.95 (0.01)	0.93, 0.96
VAS (baseline)	69.35 (1.08)	67.22, 71.47	69.50 (1.02)	67.50, 71.51
VAS (12 months)	77.16 (1.05)	75.10, 79.21	76.23 (1.01)	74.24, 78.22

### Change Scores (12 Months—Baseline)

Both groups showed improvements in HRQoL between baseline and 12 months (Table 3). For EQ-5D-5L utility, the mean change was 0.02 (SE 0.01; 95% CI 0.01 to 0.04) in the control group and 0.01 (SE 0.01; 95% CI − 0.01 to 0.03) in the intervention group. The confidence interval for the control group excluded zero, whereas the interval for the intervention group included zero. For VAS scores, the mean increase was 7.81 points (SE 1.40; 95% CI 5.06 to 10.56) in the control group and 6.72 points (SE 1.31; 95% CI 4.15 to 9.30) in the intervention group. The 95% confidence intervals for both groups excluded zero, indicating improvement over time in self-reported health status in both arms.

**Table 3:** Change in EQ-5D-5L utility and VAS (N = 582)

Outcome	Control mean (SE)	95% CI	Intervention mean (SE)	95% CI
Δ EQ-5D-5L utility	0.02 (0.01)	0.01, 0.04	0.01 (0.01)	− 0.01, 0.03
Δ VAS	7.81 (1.40)	5.06, 10.56	6.72 (1.31)	4.15, 9.30

### Between-Group Comparisons (Unadjusted)

As shown in Table 4, at baseline there were no statistically significant differences between the control and intervention arms in EQ-5D-5L utility (0.95 vs 0.94; mean difference 0.01; t = 1.09, p = 0.28) or VAS scores (69.35 vs 69.50; mean difference − 0.16; t = − 0.11, p = 0.92). At 12 months, mean EQ-5D-5L utility was higher in the control arm compared with the intervention arm (0.97 vs 0.95), with a statistically significant mean difference of 0.03 (t = 2.66, p = 0.01). However, the between-group difference in change in EQ-5D-5L utility from baseline to 12 months was not statistically significant (0.02 vs 0.01; mean difference 0.01; t = 1.09, p = 0.28). For VAS scores, no statistically significant between-group differences were observed at 12 months (77.16 vs 76.23; mean difference 0.93; t = 0.64, p = 0.52) or in change from baseline (7.81 vs 6.72; mean difference 1.09; t = 0.57, p = 0.57).

**Table 4:** Two-sample t-tests comparing arms (N = 582)

Outcome	Control mean	Intervention mean	Mean Diff (C−I)	t	df	p-value
EQ-5D-5L utility (baseline)	0.95	0.94	0.01	1.09	580	0.28
EQ-5D-5L utility (12 months)	0.97	0.95	0.03	2.66	580	0.01
Δ EQ-5D-5L utility	0.02	0.01	0.01	1.09	580	0.28
VAS (baseline)	69.35	69.50	-0.16	-0.11	580	0.92
VAS (12 months)	77.16	76.23	0.93	0.64	580	0.52
Δ VAS	7.81	6.72	1.09	0.57	580	0.57

### Primary Regression Analyses (ANCOVA)

#### EQ-5D-5L Utility at 12 Months Adjusted for Baseline Utility

In the ANCOVA model (Table 5) adjusting for baseline EQ-5D-5L utility (N = 582), allocation to the intervention arm was associated with a 0.02 lower 12-month EQ-5D-5L utility score compared to the control arm (β = − 0.02, SE = 0.01, t = − 2.47, p = 0.01; 95% CI: − 0.04 to − 0.00). Baseline EQ-5D-5L utility was positively associated with 12-month utility (β = 0.21, SE = 0.04, t = 5.79, p < 0.001; 95% CI: 0.14 to 0.28), indicating that higher baseline utility predicted higher follow-up utility. The overall model was statistically significant (F(2, 579) = 20.50, p < 0.001) and explained approximately 7% of the variance in 12-month EQ-5D-5L utility (R2 = 0.07).

**Table 5:** ANCOVA predicting 12-month EQ-5D-5L utility (N = 582)

Predictor	Coefficient	SE	t	p-value	95% CI
Intervention (vs control)	− 0.02	0.01	− 2.47	0.01	− 0.04, − 0.00
EQ-5D-5L utility (baseline)	0.21	0.04	5.79	0.00	0.14, 0.28
Constant	0.78	0.03	22.31	0.00	0.71, 0.84

Model summary: R2 = 0.07; F(2, 579) = 20.50; N = 582

#### VAS at 12 Months Adjusted for Baseline VAS

Table 6, in the ANCOVA model adjusting for baseline VAS, there was no statistically significant difference in 12-month VAS scores between the intervention and control groups (β = − 0.95, SE = 1.44, p = 0.51). The estimated difference indicates that, after controlling for baseline VAS, participants in the intervention arm had VAS scores that were on average 0.95 points lower than those in the control arm, however, the 95% confidence interval (-3.79 to 1.88) included zero. Baseline VAS was a statistically significant predictor of 12-month VAS (β = 0.15, SE = 0.04, p < 0.001), indicating that higher baseline self-rated health was associated with higher VAS scores at follow-up. The model explained a small proportion of variance in 12-month VAS scores (R2 = 0.02), although the overall model was statistically significant (F(2, 579) = 7.21, p < 0.001).

**Table 6:** ANCOVA predicting 12-month VAS (N = 582)

Predictor	Coefficient	SE	t	p-value	95% CI
Intervention (vs control)	− 0.95	1.44	− 0.66	0.51	− 3.79, 1.88
VAS (baseline)	0.15	0.04	3.74	0.00	0.07, 0.23
Constant	66.71	2.98	22.39	0.00	60.86, 72.57

Model summary: R2 = 0.02; F(2, 579) = 7.21; N = 582

### Fully Adjusted Regression Analyses (Sociodemographic Covariates)

#### Adjusted Model: EQ-5D-5L Utility at 12 Months

In the fully adjusted model (N = 552), intervention status remained independently associated with 12-month EQ-5D-5L utility after controlling for baseline utility and sociodemographic covariates (Table 7). Participants in the intervention arm had, on average, a 0.03 lower EQ-5D-5L utility score at 12 months compared with the control arm (β = − 0.03; 95% CI: − 0.05 to − 0.01; p < 0.01). Baseline EQ-5D-5L utility was positively associated with 12-month utility (β = 0.20; 95% CI: 0.13 to 0.28; p < 0.01). Age was marginally negatively associated with 12-month utility (β = − 0.00; 95% CI: − 0.00 to − 0.00; p = 0.05). Female participants had lower 12-month EQ-5D-5L utility scores compared with males (β = − 0.03; 95% CI: − 0.05 to − 0.00; p = 0.04). Education level, employment status, household size, number of children, and housing characteristics (electricity, flush toilet, piped water) were not statistically significantly associated with 12-month EQ-5D-5L utility in the adjusted model (all p > 0.05). The model explained approximately 10% of the variation in 12-month EQ-5D-5L utility (R2 = 0.10; adjusted R2 = 0.08; F(11, 540) = 5.20).

**Table 7:** Fully adjusted regression predicting 12-month EQ-5D-5L utility (N = 552)

Predictor	Coefficient	SE	t	p-value	95% CI
Intervention (vs Control)	− 0.03	0.01	− 2.99	0.00	− 0.05, − 0.01
EQ-5D-5L utility (baseline)	0.20	0.04	5.67	0.00	0.13, 0.28
Age	− 0.00	0.00	− 1.97	0.05	− 0.00, − 0.00
Female (vs Male)	− 0.03	0.01	− 2.03	0.04	− 0.05, − 0.00
Secondary (Grade 1–7) (vs Primary (Grade 8–12))	− 0.01	0.01	− 0.75	0.45	− 0.04, 0.02
Employed (vs Not working)	0.00	0.01	0.44	0.66	− 0.02, 0.02
Household size	0.00	0.00	0.27	0.78	− 0.00, 0.01
Number of children	0.00	0.00	1.03	0.31	− 0.00, 0.00
Electricity in house (Yes)	0.03	0.02	1.54	0.13	− 0.01, 0.06
Flush toilet (Yes)	− 0.02	0.02	− 0.94	0.35	− 0.06, 0.02
Piped water (Yes)	− 0.00	0.02	− 0.11	0.92	− 0.04, 0.04
Constant	0.85	0.05	16.65	0.00	0.75, 0.96

Model summary: R2 = 0.10; adjusted R2 = 0.08; F(11, 540) = 5.20; N = 552

#### Adjusted Model: VAS at 12 Months

In the fully adjusted model (N = 552) (Table 8), there was no statistically significant difference in 12-month VAS scores between the intervention and control groups (β = − 1.25, 95% CI: − 4.15 to 1.64; p = 0.40). Baseline VAS score was positively associated with 12-month VAS (β = 0.14, 95% CI: 0.06 to 0.22; p < 0.001), indicating that higher self-rated health at baseline predicted higher self-rated health at follow-up. Female participants reported significantly lower VAS scores at 12 months compared with males (β = − 5.09, 95% CI: − 8.92 to − 1.25; p = 0.01). Access to electricity in the household was associated with higher VAS scores (β = 8.27, 95% CI: 3.26 to 13.29; p < 0.001), while having a flush toilet was associated with lower VAS scores (β = − 6.74, 95% CI: − 12.56 to − 0.92; p = 0.02). Age, education level, employment status, household size, number of children, and piped water access were not significantly associated with 12-month VAS scores (all p > 0.05). The overall model explained 7% of the variance in 12-month VAS scores (R2 = 0.07; adjusted R2 = 0.05; F(11, 540) = 3.44).

**Table 8:** Fully adjusted regression predicting 12-month VAS (N = 552)

Predictor	Coefficient	SE	t	p-value	95% CI
Intervention (vs Control)	− 1.25	1.47	− 0.85	0.40	− 4.15, 1.64
VAS (baseline)	0.14	0.04	3.33	0.00	0.06, 0.22
Age	− 0.10	0.09	− 1.10	0.27	− 0.29, 0.08
Female (vs Male)	− 5.09	1.95	− 2.60	0.01	− 8.92, − 1.25
Secondary (vs Primary)	0.87	2.01	0.43	0.67	− 3.09, 4.82
Employed (vs Not working)	2.10	1.58	1.34	0.18	− 0.99, 5.20
Household size	0.26	0.41	0.64	0.53	− 0.55, 1.07
Number of children	0.00	0.00	0.06	0.95	− 0.01, 0.01
Electricity in house (Yes)	8.27	2.55	3.24	0.00	3.26, 13.29
Flush toilet (Yes)	− 6.74	2.96	− 2.28	0.02	− 12.56, − 0.92
Piped water (Yes)	2.83	3.03	0.94	0.35	− 3.11, 8.77
Constant	69.89	6.53	10.70	0.00	57.06, 82.73

Model summary: R2 = 0.07; adjusted R2 = 0.05; F(11, 540) = 3.44; N = 552

### Standardised Effect Size (EQ-5D-5L at 12 Months)

At 12 months, the mean EQ-5D-5L utility score was 0.03 units higher in the control group compared with the intervention group (Table 9). Using the pooled standard deviation of 0.11, this corresponds to a standardised effect size (Cohen’s d) of 0.22. This magnitude is consistent with a small effect size.

**Table 9:** Effect size for 12-month EQ-5D-5L utility (N = 582)

Metric	Value
Mean difference (control−intervention)	0.03
Pooled SD	0.11
Cohen’s d	0.22

### Harms

No adverse events or unintended harms attributable to the intervention were reported during the study period.

## Discussion

This study assessed whether a 12-month SMS-based mHealth intervention improved HRQoL among adults living with HIV and comorbid hypertension in primary care in the Western Cape. Overall, HRQoL levels were high at baseline, and changes over 12 months were modest. In this context, the key message is not one of substantial HRQoL gains, but rather that the intervention did not translate into measurable improvements in overall EQ-5D-5L utility or VAS relative to usual care. This pattern is consistent with evidence from mHealth evaluations in chronic disease management, where effects on quality of life are often small and heterogeneous, and where interventions more commonly influence proximal behaviours (e.g., adherence) than downstream HRQoL outcomes [32–35, 42]. The demographic profile predominantly middle-aged women aligns with other work in sub-Saharan Africa, and the burden of pain or discomfort and anxiety or depression reported in similar populations is well described in the HIV and multimorbidity literature [11, 37, 43–45].

Although unadjusted follow-up EQ-5D-5L utility was higher in the control arm, and ANCOVA models suggested a small negative association between intervention allocation and 12-month EQ-5D-5L utility, the magnitude of this difference was small (Cohen’s d = 0.22). This suggests that even where statistical differences are observed, the practical impact on overall health utility is limited. One plausible explanation is that the trial population started with very high utility scores, leaving little scope for detectable improvement an important ceiling effect issue in EQ-5D-based analyses in relatively stable chronic care cohorts [14]. Where baseline values cluster near “full health,” small fluctuations (including those driven by measurement error, temporary symptoms, or regression to the mean) can dominate the observed between-group differences, without reflecting meaningful differences in lived wellbeing.

The contrast between EQ-5D-5L utility and the VAS is also informative. The VAS captures a global self-assessment of health that may be more responsive to subjective wellbeing, confidence in self-management, or day-to-day perceptions than the EQ-5D descriptive system [46, 47]. In this study, VAS outcomes did not differ between arms in adjusted models, suggesting that the SMS programme did not improve perceived overall health relative to usual care. This aligns with broader evidence that low-intensity, one-way text messaging while scalable may be insufficient to shift patient-reported outcomes unless combined with more interactive support, tailoring, or clinical feedback loops [34, 48, 49]. Interventions that incorporate bidirectional communication, adaptive content, or linkage to care teams may be better positioned to influence psychosocial pathways (motivation, self-efficacy, distress) that plausibly mediate HRQoL change.

Several design features may help explain the modest effects. First, the intervention relied on standardised, one-way reminders, which limits personalisation and responsiveness to individual barriers. Second, HRQoL is a relatively distal outcome for an adherence-focused SMS intervention, improvements in HRQoL may require either larger clinical effects (e.g., better blood pressure control and fewer symptoms) or broader psychosocial intervention components. Third, the EQ-5D-5L may not capture changes that matter for this population such as stress related to multimorbidity management, financial strain, or treatment burden particularly when changes are subtle or domain-specific [46, 47]. Together, the results support a cautious interpretation, SMS-based support may be helpful as a low-cost adjunct within integrated HIV–NCD care, but it should not be expected to generate meaningful improvements in overall HRQoL without added components that increase intensity, responsiveness, or linkage to broader support.

From a policy perspective, SMS programmes remain attractive because they are inexpensive and scalable, and they can be layered onto existing ART and chronic care platforms with limited additional workforce requirements. However, this study suggests that if improving HRQoL is a primary objective, programme design should move beyond reminder-only approaches toward more patient-centred messaging strategies, including tailoring, bidirectional communication, and mechanisms for escalation or referral when symptoms or adherence problems are identified. Embedding SMS tools within routine systems (e.g., chronic care registers and differentiated service delivery models) may also improve effectiveness by ensuring continuity with clinic-based care and follow-up.

### Strengths and Limitations

A key strength of this study is its focus on a dual-diagnosis population people living with HIV and comorbid hypertension for whom evidence on HRQoL impacts of mHealth interventions remains limited. The use of both the EQ-5D-5L utility index and the VAS provides complementary perspectives on HRQoL, allowing comparison between a preference-based measure suitable for economic evaluation and a global self-rating of health. The analytic approach used ANCOVA models that appropriately adjusted for baseline HRQoL and were extended to fully adjusted specifications including sociodemographic and household characteristics, improving precision and reducing measured confounding.

Several limitations should be considered. First, although models adjusted for available sociodemographic and household covariates, residual confounding cannot be excluded. Important time-varying or unmeasured factors.

such as pregnancy, changes in housing or employment status, incident comorbidities, medication side effects, mental health status, or changes in clinical control of HIV or hypertension were not explicitly modelled and may have influenced HRQoL trajectories over follow-up. As such, the observed differences in EQ-5D-5L utility between groups should be interpreted cautiously. Second, the follow-up period of 12 months may be insufficient for HRQoL changes to manifest, particularly if clinical improvements accrue slowly or require sustained behavioural engagement. Outcomes were self-reported, which may introduce reporting bias and day-to-day variability.

Third, the EQ-5D-5L, while widely validated, may not fully capture dimensions of quality of life that are particularly salient for people living with HIV. Domains such as stigma, disclosure concerns, social support, medical mistrust, treatment burden, and medication side effects are not directly measured within the EQ-5D descriptive system. This may limit its sensitivity to detect meaningful changes in psychosocial wellbeing in this population. In addition, the high baseline utility scores observed in this cohort raise the possibility of ceiling effects, which may reduce the instrument’s ability to detect small but clinically relevant differences over time.

Finally, utilities were derived using the Ugandan [37], EQ-5D-5L value set because a South African tariff was not available at the time of analysis. Although Uganda represents a contextually relevant regional alternative, the absence of a country-specific value set may affect absolute utility estimates and comparability with future South African studies. Future research should test more interactive mHealth designs such as bidirectional SMS, tailored and adaptive messaging, or app-enabled support combined with stronger integration into clinical management (e.g., blood pressure monitoring feedback, nurse follow-up triggers). Longer follow-up, incorporation of objective clinical outcomes, and use of HIV-specific or multidimensional HRQoL instruments may help clarify the pathways through which mHealth interventions influence wellbeing in multimorbid populations.

## Conclusion

In adults living with HIV and comorbid hypertension in primary care, a 12-month SMS-based intervention did not improve HRQoL relative to usual care, and any differences in EQ-5D-5L utility at follow-up were small in magnitude. These findings suggest that reminder-only SMS approaches may be insufficient to generate meaningful HRQoL gains in multimorbidity care, particularly where baseline HRQoL is already high. Low-cost messaging programmes may still have value as scalable adjuncts within integrated HIV–NCD services, but more interactive and patient-centred designs are likely required if the goal is to improve quality of life rather than support adherence alone.

## Data Availability

The datasets analyzed during the current study are not publicly available due to participant confidentiality but may be made available from the corresponding author upon reasonable request, subject to institutional approvals and data-sharing agreements.

## Code Availability

Statistical analysis code used in this study may be made available from the corresponding author upon reasonable request.

## Protocol Availability

The trial protocol and statistical analysis plan are available from the corresponding author upon reasonable request, subject to institutional approvals.
